# External Validation of a Predictive Model of Urethral Strictures for Prostate Patients Treated With HDR Brachytherapy Boost

**DOI:** 10.3389/fonc.2020.00910

**Published:** 2020-06-11

**Authors:** Vanessa Panettieri, Tiziana Rancati, Eva Onjukka, Martin A. Ebert, David J. Joseph, James W. Denham, Allison Steigler, Jeremy L. Millar

**Affiliations:** ^1^Alfred Health Radiation Oncology, Alfred Hospital, Melbourne, VIC, Australia; ^2^Medical Imaging and Radiation Sciences, Monash University, Clayton, VIC, Australia; ^3^Prostate Cancer Program, Scientific Directorate, Fondazione IRCCS Istituto Nazionale dei Tumori, Milan, Italy; ^4^Medical Radiation Physics and Nuclear Medicine, Karolinska University Hospital, Stockholm, Sweden; ^5^Radiation Oncology, Sir Charles Gairdner Hospital, Perth, WA, Australia; ^6^School of Physics, Mathematics and Computing, University of Western Australia, Perth, WA, Australia; ^7^5D Clinics, Claremont, WA, Australia; ^8^GenesisCare, Subiaco, WA, Australia; ^9^School of Surgery, University of Western Australia, WA, Australia; ^10^School of Medicine and Public Health, University of Newcastle, Newcastle, NSW, Australia; ^11^Central Clinical School, Monash University, Melbourne, VIC, Australia

**Keywords:** NTCP, HDR brachytherapy, urethra, predictive modeling, prostate cancer

## Abstract

**Purpose:** For prostate cancer treatment, comparable or superior biochemical control was reported when using External-Beam-Radiotherapy (EBRT) with High-Dose-Rate-Brachytherapy (HDRB)-boost, compared to dose-escalation with EBRT alone. The conformal doses produced by HDRB could allow further beneficial prostate dose-escalation, but increase in dose is limited by normal tissue toxicity. Previous works showed correlation between urethral dose and incidence of urinary toxicity, but there is a lack of established guidelines on the dose constraints to this organ. This work aimed at fitting a Normal-Tissue-Complication-Probability model to urethral stricture data collected at one institution and validating it with an external cohort, looking at neo-adjuvant androgen deprivation as dose-modifying factor.

**Materials and Methods:** Clinical and dosimetric data of 258 patients, with a toxicity rate of 12.8%, treated at a single institution with a variety of prescription doses, were collected to fit the Lyman–Kutcher–Burman (LKB) model using the maximum likelihood method. Due to the different fractionations, doses were converted into 2 Gy-equivalent doses (α/β = 5 Gy), and urethral stricture was used as an end-point. For validation, an external cohort of 187 patients treated as part of the TROG (Trans Tasman Radiation Oncology Group) 03.04 RADAR trial with a toxicity rate of 8.7%, was used. The goodness of fit was assessed using calibration plots. The effect of neo-adjuvant androgen deprivation (AD) was analyzed separating patients who had received it prior to treatment from those who did not receive it.

**Results:** The obtained LKB parameters were TD50 = 116.7 Gy and *m* = 0.23; *n* was fixed to 0.3, based on numerical optimization of the likelihood. The calibration plot showed a good agreement between the observed toxicity and the probability predicted by the model, confirmed by bootstrapping. For the external validation, the calibration plot showed that the observed toxicity obtained with the RADAR patients was well-represented by the fitted LKB model parameters. When patients were stratified by the use of AD TD50 decreased when AD was not present.

**Conclusions:** Lyman–Kutcher–Burman model parameters were fitted to the risk of urethral stricture and externally validated with an independent cohort, to provide guidance on urethral tolerance doses for patients treated with a HDRB boost. For patients that did not receive AD, model fitting provided a lower TD50 suggesting a protective effect on urethra toxicity.

## Introduction

In the treatment of unfavorable prostate cancer, several studies have shown that the use of High-Dose-Rate Brachytherapy (HDRB) as a boost in combination with External Beam Radiotherapy (EBRT) provides biochemical control and prostate-cancer specific survival comparable or superior to dose-escalation with EBRT alone (1–6). These results are in line with findings suggesting that prostate cancer tends to respond similarly to late reacting tissues to dose fractionation schedules, consistent with lower α/β ratio (7, 8). The conformal doses provided by HDRB could potentially allow further beneficial dose-escalation due to their excellent organs-at-risk (OARs) sparing. However, concerns have been raised regarding the potential risk of acute and late urethral toxicity, in particular urethral stricture, which has been reported by several authors in rates up to 30% (9–11). Causes for urethral strictures have been investigated and contradictory findings are reported in the literature with reports showing correlation between urethral dose and incidence of urinary toxicity (10, 12), and others instead reporting no significant correlations (2, 13, 14).

Due to the variety of fractionation regimens used for HDRB boost treatments in different centers, ranging from multiple fractions to monotherapy (9), it is still hard to compare practices and related toxicity results. Additionally, follow-up time tends to vary ranging between 2 and over 5 years (2, 10, 13). For this reason, there is no consensus on the dose constraints for urethral doses (15–17), and often limits are decided in each institution based on experience of the practitioners. In-depth analyses of the dose-effect relationships have been performed for the bladder and urethral toxicity mainly in the context of EBRT to gain understanding of the potential effect of increasing dose per fraction on the main OARs, following the increase in the use of hypofractionation in prostate radiotherapy treatments (18–20). A small number of studies have also looked at Normal Tissue Complication Probability (NTCP) for the urethra, but in all cases, they have highlighted that parameters for the most used NTCP models, such as the relative seriality or the Lyman–Kutcher–Burman model, were not available, and have assumed that the urethra had a similar response as organs such the esophagus (21, 22).

Using the long term data and experience accumulated in our department in treating prostate cancer patients with HDRB boost the purpose of this work has been to establish NTCP model parameters specific for the urethra by fitting a normal tissue toxicity curve on urethral stricture data recorded in our institution. This curve has then been validated with an independent external cohort, in order to provide general applicability and a tool to guide treatment design and fractionation selection criteria.

## Materials and Methods

### Model Fitting

#### Patients and Clinical Data

Clinical and three dimensional (3D) treatment planning data of 258 patients treated at Alfred Health Radiation Oncology (AHRO) from 2001 to 2013 were retrospectively collected for this analysis. These 258 patients were selected as a subset of a larger group of more than 500 patients treated at our institution, receiving a curative regimen that included a boost of HDRB, in combination with EBRT, since they had complete retrievable 3D planning and associated toxicity information with at least 4 years of follow-up. Most patients were classified in the intermediate and high risk group, and details of the CT-planning based treatment technique are presented in previous publications (10, 23). In summary, for patients treated before 2006 metal needles, replaced by plastic needles for patients treated after 2006, were inserted transperineally using ultrasound guidance. Before 2005, patients were not replanned in subsequent days, then until 2008 only if a second CT-simulator scan showed a superior-inferior displacement of the needles of more than 1 cm. As of 2008 for all patients, a new CT scan and plan is performed on the second day. All patients received an EBRT dose of 46–50 Gy in 2 Gy per fraction. For the HDRB boost, a variety of fractionations regimens were used to treat the patients over the years (Table 1), but all patients were treated in 2 consecutive days, with the patients treated with three fractions having two fractions on the 2nd day.

**Table 1:** AHRO HDRB boost patients' characteristics including number of patients (no. of patients), HDRB physical, and biological prescription dose (respectively, Brachytherapy Prescription dose-physical and equivalent), toxicity rate, mean, and median time to stricture (%), patient who had received Neo-Adjuvant Androgen Deprivation and age.

	**Group 1**	**Group 2**	**Group 3**	**Group 4**	**Total**
No of patients	131	117	8	2	258
Brachytherapy Prescription dose (physical dose, Gy)	18 Gy in 3 fractions	19 Gy in 2 fractions	17 Gy in 2 fractions	10 and 6 Gy in 2 fractions	
External Beam Prescription dose (physical dose, Gy)	46 Gy in 23 fractions	46 Gy in 23 fractions	46 Gy in 23 fractions	46 Gy in 23 fractions	
Brachytherapy Prescription dose (2 Gy equivalent dose, α/β = 5 Gy)	28.3 Gy	39.4 Gy	32.8 Gy	30.8 Gy	
Total dose EBRT + HDRB (2 Gy equivalent dose, α/β = 5 Gy)	74.3 Gy	85.4 Gy	78.8 Gy	76.8 Gy	
Toxicity rate at 4 years (%)	6.9%	20.5%	0%	0%	12.8%
Mean time to stricture (years)	3.6	2.1	Not applicable	Not applicable	
Median time to stricture (years)	3.0	1.4	Not applicable	Not applicable	
Adjuvant androgen deprivation (no of patients)	118	113	8	2	241
Mean age (years)	65.4	66.3	66.1	65	65.7

*For EBRT the 46 in 2 Gy per fraction prescription is shown as only 1 patient in the whole cohort had 50 in 2 Gy per fraction*.

**Figure 1:**
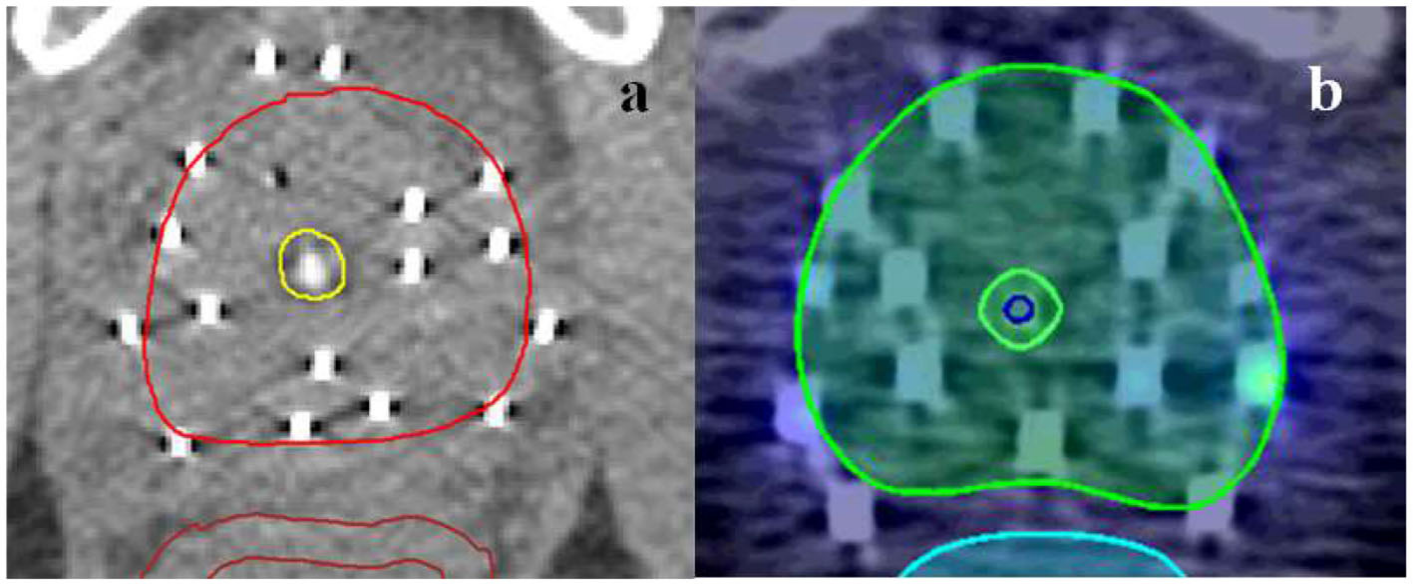
Urethra contouring characteristics for AHRO **(a)** and RADAR **(b)** patient.

For all patients, clinical, demographic, and toxicity data were extracted from our institutional prospective brachytherapy database BrachyNET. All patients had a review after 6, 12, 24 months and every year until 10 years after the HDRB implant, and no patient was lost to follow-up. At each review, patients completed the Expanded Prostate Cancer Index Composite (EPIC-26) form (24), and rectal and urethral toxicity information was collected. In terms of urethral toxicity, a stricture was recorded if the patient underwent a surgical procedure for a stricture (dilatation or urethrotomy). In this work, the end-point was chosen to be the time of the first urethrotomy, with a follow-up cut off time of 4 years, and the average stricture rate was 12.8%. Among the clinical parameters, age, and the use of neo-adjuvant androgen deprivation (AD) were also collected (Table 1). In the HDRB plan, the urethra was contoured by the Radiation Oncologist (RO) around the external diameter of a 22-Fr gauge three-way indwelling urinary catheter as a solid structure from typically 1 cm below the apex to the bladder base (Figure 1a) considering the specific anatomy of each patient to include the mucosal wall. OAR doses were limited using departmental guidelines based mainly on the GEC-ESTRO recommendations (15). For the Planning Target Volume (PTV): *D*_90%_ > 100% (at least 100% of prescribed dose covering 90% of PTV), *V*_100%_ > 95% (i.e., 95% of PTV receiving at least 100% of the prescription dose), *V*_150%_ = 15–32% (i.e., 150% of the prescription dose to 15–32% of the PTV), *V*_200%_ = 5–9% (i.e., 200% of the prescription dose to 5–9% of the PTV). For the OARs: urethra *D*_10%_ <110% (i.e., 10% of urethra receiving no more than 110% of the prescription dose), and rectal wall *D*_2cc_ <66% (i.e., 2 cc of rectal wall receiving no more than 66% of the prescription dose).

#### Dosimetric Data

Due to the long time period for patient treatment included in this study the AHRO HDRB patient treatment plans were originally calculated either in the Plato (Nucletron) or in Oncentra treatment planning system (Elekta). In order to limit differences due to different Dose Volume Histogram (DVH) estimates, all plans were de-identified and re-imported in Oncentra, and DVHs were recalculated and exported. Since the patients were treated with four different fractionation regimens, and due to the inhomogeneous dose in the urethra, each fraction's physical doses were converted into equivalent doses in 2 Gy per fraction (EQD_2_) considering an α/β ratio of 5 Gy, as previously used by Gloi and Buchanan (22) (of note equivalent doses for late effects to normal tissues are of interest in the frame of this work). Due to the conformal nature of the EBRT plan, it was assumed that for all patients the urethra had received the full EBRT prescription dose of 46–50 Gy in 2 Gy per fraction. Converted prescription doses for the brachytherapy boost are shown in Table 1.

#### Determination of the Model Parameters

The Lyman–Kutcher–Burman model (LKB) was used in this analysis (25, 26), and the dose-response curve plotted as a function of the equivalent uniform dose (EUD). The determination of the best estimate of the model parameters was done by fitting clinical and dosimetric data using the maximum likelihood method as previously described (27, 28), using MatlabR2018 (Mathworks). Due to the small urethral volumes involved, initially a numerical optimization of the likelihood function was performed to establish a volume effect parameter (*n*) value descriptive of the relationship between urethral “architecture” and the considered toxicity endpoint in the available dataset. Then this value was fixed, and TD50 (Gy) (EUD that causes 50% probability of toxicity) and *m* (slope of the response curve at TD50) were fitted. As the most recent patients were rescanned and replanned on the 2nd day of treatment, EUD from day 1 and 2 were considered in the model.

Internal validation was performed by bootstrapping the original dataset 1,000 times as previously described (29), and recalculating the model parameters. Results from the bootstrap procedure were also used to define confidence intervals for best-fit parameters: a 68% confidence interval was calculated as the range 16th−84th percentiles of the distribution of the parameter values obtained through bootstrap, while a 95% confidence interval was calculated as the range 2.5th−97.5th percentiles of the same distribution.

Goodness of fit was determined by using a model calibration plot to establish the relationship between the observed and predicted probability. Due to the binary nature of the stricture data (yes/no) the observed probabilities were obtained by dividing the 258 patients studied into four dose-bin groups and determining the corresponding rate of toxicity of each group. These observed rates were then plotted against those predicted by the model and a trend line derived. This line was then compared against the identity line which represents a perfect prediction (30). Calibration plot was established for the model fitted with the original AHRO data (apparent calibration line). Bootstrapping was employed to determine optimism and optimism-corrected performance (calibration line after correction for optimism) was then calculated as described by Steyerberg (31).

The discriminative ability of the model, that is, the ability to distinguish patients with different outcomes, was also evaluated with the area under the receiver operating characteristic curve (AUC).

### External Model Validation

Data from a second cohort of 187 patients from a different institution treated as part of the TROG (Trans Tasman Radiation Oncology Group) 03.04 RADAR trial (32) were collected. For this group of patients, the HDRB prescription dose was 19.5 Gy in three fractions [corresponding to EQD_2_ (α/β = 5 Gy)], the stricture rate at 8.6% was comparable to AHRO patients, and all patients had ~5 months of AD prior to radiotherapy, as part of a randomized total of 6 or 18 months of AD. The urethral toxicity end-point was considered to be equivalent to the one chosen for the AHRO patients, as the time of the first urethrotomy. The RADAR cohort was also treated with EBRT doses of 46 in 2 Gy fractions. For this group urethral structures were initially contoured by the RO as the visible lumen of the urinary catheter (Figure 1b, blue contour) and, then, these original contours were expanded on average 2 mm in the anterior-posterior and left-right direction and modified in the superior-inferior direction to be similar to the AHRO contours (Figure 1b, yellow contour). An expansion was chosen in order to preserve the variability in contours due to the RO outlines and provided urethral volumes on average equivalent to those obtained in the AHRO patients (respectively, expanded RADAR 1.5 cm^3^ and AHRO 1.4 cm^3^).

Both structures' DVHs (RADAR original and expanded) and associated clinical data were used to externally validate the LKB parameters obtained with the AHRO cohort. Model calibration, as described above, was used to establish agreement between the AHRO model estimated probabilities and RADAR observed stricture rates.

### Effect of Clinical Covariates As Dose-Modifying Factors

For the AHRO patients, the effect of using AD on the model parameters was also investigated. The *n* and *m* value of the LKB model parameters were fixed and the fit was re-done separating the patients with (241/258) and without AD (17/258) to obtain two different TD50s as proposed by Peeters et al. (33).

## Results

### LKB Model Parameters

**Figure 2:**
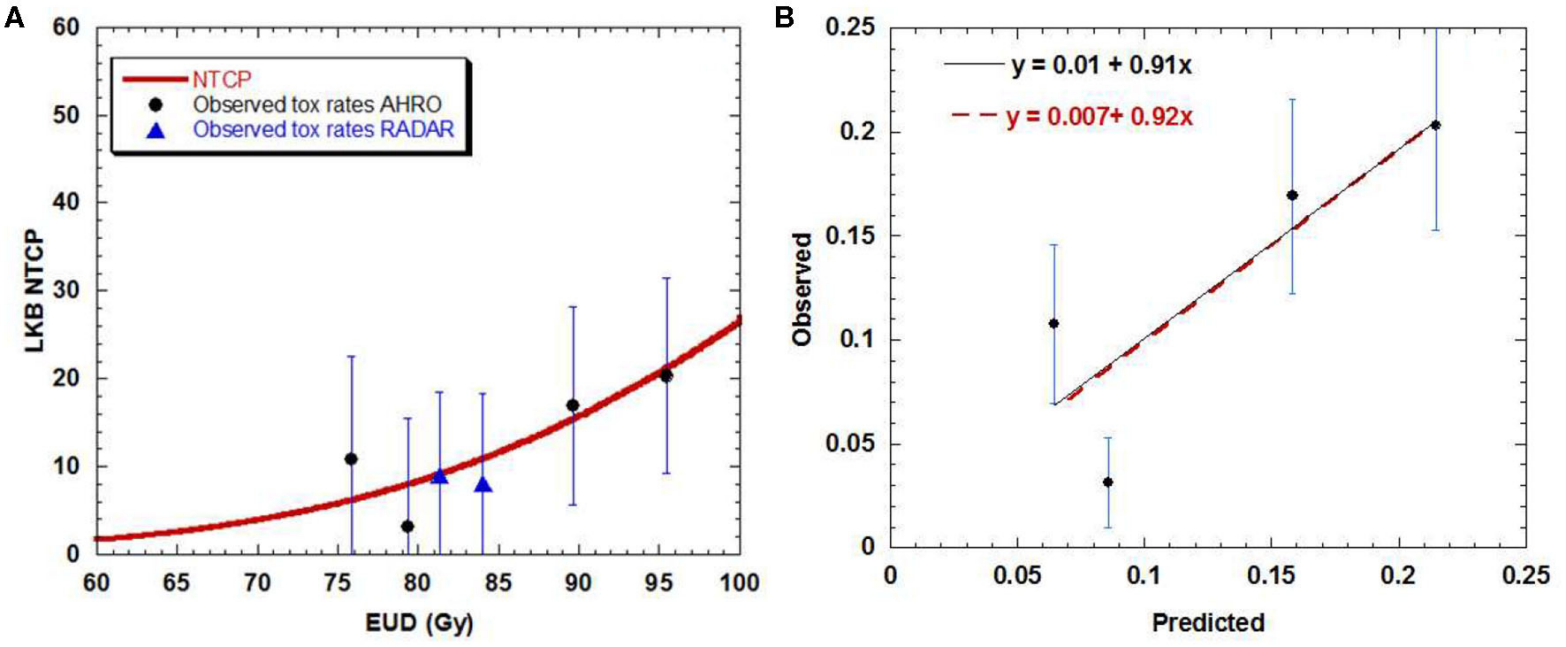
**(A)** Dose-volume response curve obtained with the best estimated parameters for the LKB model for urethral stricture. Solid black circles represent the AHRO observed toxicity rates with corresponding error. The blues triangles represent the RADAR patients. **(B)** Calibration (predicted vs. observed) curves obtained by using the AHRO LKB model and data (red dotted line, apparent calibration line; black continuous line, calibration line after correction for optimism).

**Figure 3:**
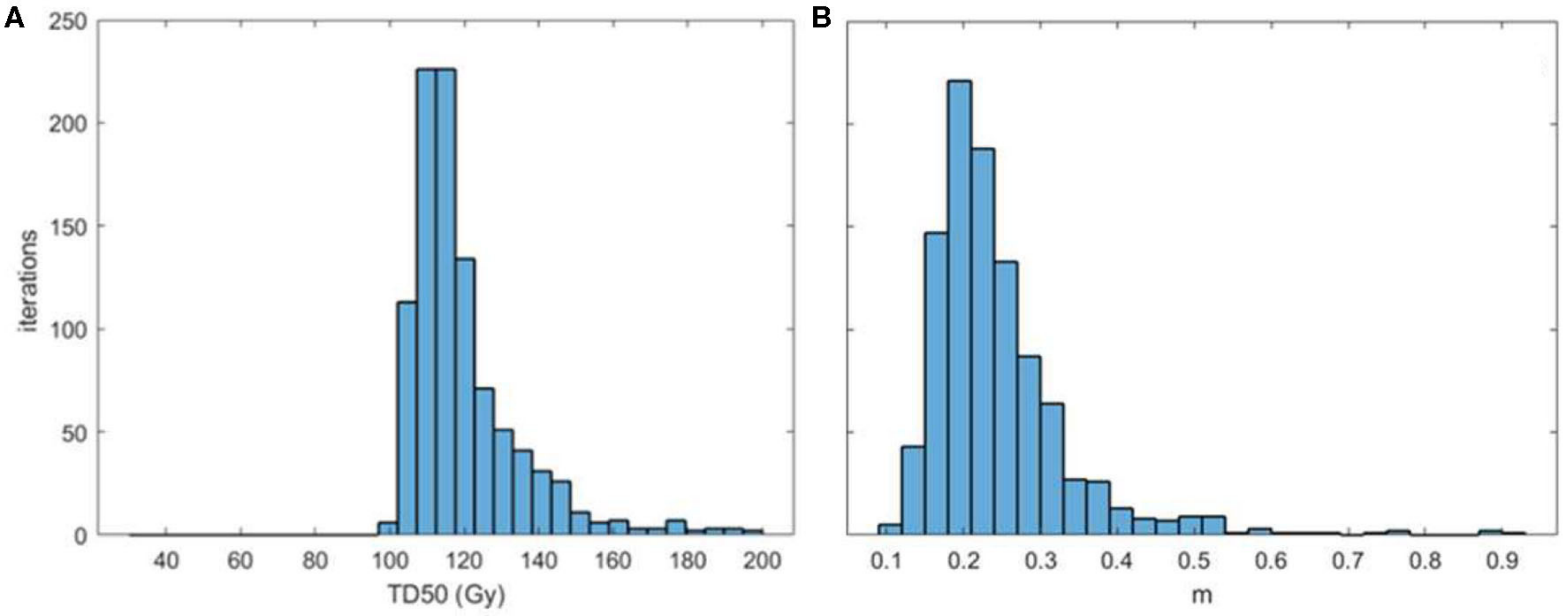
Distribution of TD50 (Gy) **(A)** and *m* **(B)** parameters obtained with bootstrapping.

For the AHRO patients, the urethral stricture prediction for the complete treatment (HDRB + EBRT) was modeled by means of a sigmoid function of EUD (Figure 2A). The numerical optimization of the likelihood showed a maximum for *n* = 0.3. The remaining best fitted parameters were found to be TD50 = 116.7 Gy (68% confidence interval, 108.3–134.1 Gy), *m* = 0.23 (68% confidence interval, 0.17–0.31; Table 2). The AUC of the development population was 0.64. Figure 3 reports the distribution of TD50 (Gy) and *m* parameters obtained with bootstrapping.

**Table 2:** LKB model parameters obtained fitting the original AHRO data (all cohort), with bootstrapping, corresponding Confidence Intervals (CI) and when the cohort was separated by the use or not of Androgen Deprivation (AD).

	**TD50 (Gy)**	**m**	**n**
AHRO best fit	116.7	0.23	0.3
AHRO Bootstrapping median	116.5	0.23	0.3
AHRO Bootstrapping 68% CI	108.2–134	0.17–0.31	
AHRO Bootstrapping 95% CI	104.2–218.7	0.14–0.51	
AHRO with AD	118.2	0.23	0.3
AHRO without AD	104.9	0.23	0.3

The LKB NTCP curve was obtained and compared with the AHRO observed data (Figure 2A). The calibration plot confirmed the agreement between the observed probability of the outcome and the probability predicted by the model, as the trend line between the data was close to the identity line (Figure 2B), with calibration in the large = 0.007 and slope = 0.92, *R*^2^ = 0.71 for apparent calibration and calibration in the large = 0.01 and slope = 0.91 after correction for optimism.

### External Validation of the Model

**Figure 4:**
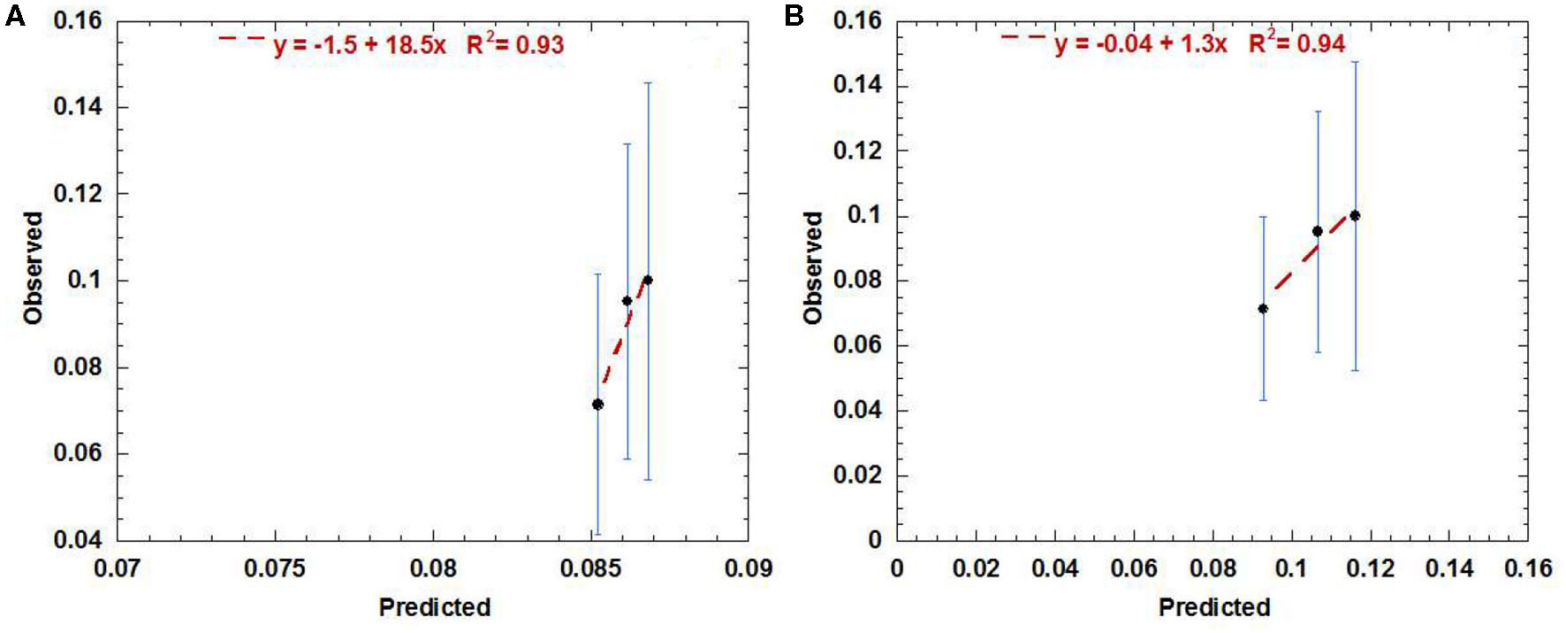
Calibration (predicted vs. observed) curve obtained by using the AHRO LKB model for the RADAR data with original contours **(A)** and the expanded contours **(B)**.

The external validation performed using the urethra data exported from the RADAR cohort gave the best agreement with the AHRO prediction model when the urethra contours were expanded to be similar to AHRO's contours (calibration in the large = −0.04 and calibration slope = 1.3, *R*^2^ = 0.94). As shown in Figure 4B, poorer calibration was found when using the original contours (calibration in the large = −1.5 and calibration slope = 18.5, *R*^2^ = 0.93; Figure 4A).

Looking at the dose-response curve (Figure 2A), consistency was found between the RADAR observed toxicity rates and the AHRO LKB model, confirming that the RADAR toxicity was well-represented by the estimated LKB model parameters.

### Effect of Using Neo-Adjuvant Androgen Deprivation

**Figure 5:**
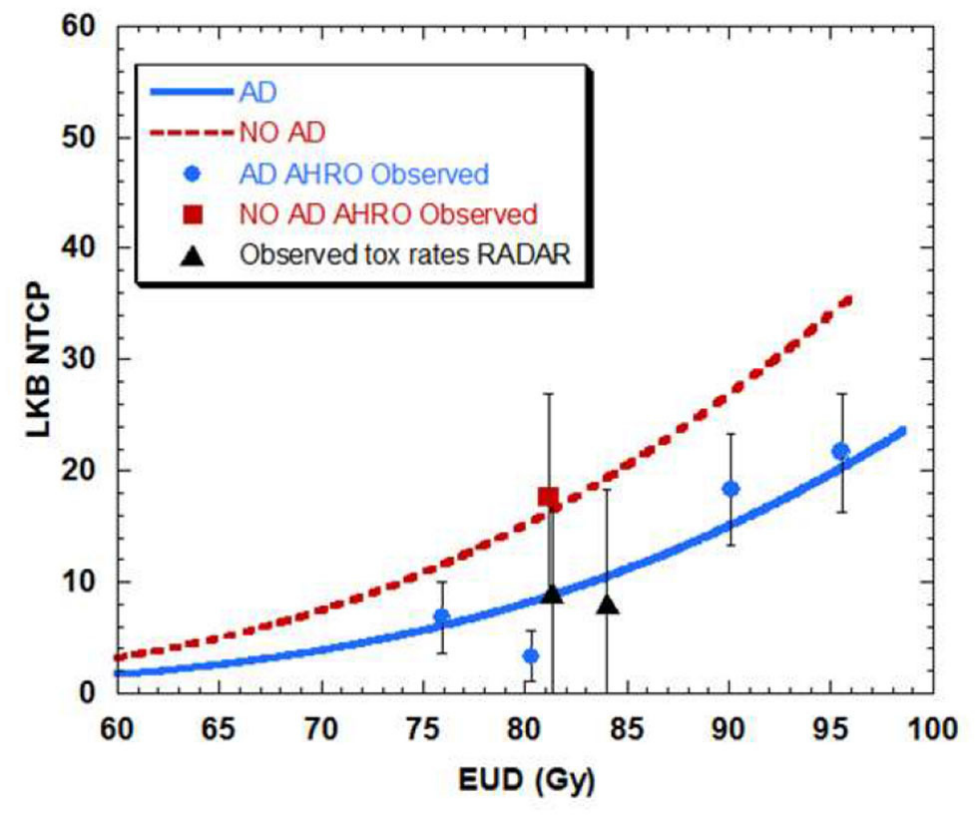
Dose-volume response curve obtained with the best estimated parameters for the LKB model for urethral stricture for patients that had neo-adjuvant androgen deprivation (AD-blue) as opposed to patient that did not have AD (NO AD-red).

When separating AHRO patients that received AD from those that did not receive it, results showed a decrease of around 13 Gy in the TD50 (Gy) for patients who did not receive AD, suggesting a protective effect of AD (Table 2, Figure 5).

## Discussion

Interest in understanding the nature of long term side effects in OARs produced by prostate radiotherapy has grown due to the increase in utilization of hypofractionated regimens in EBRT (34–36). Of particular concern is the risk of urethral stricture which generally requires surgical intervention to be resolved. Guidelines for urethral dose constraints are still sparse due to the fact that urethra contouring has only recently being considered for such techniques and correlation with dose, and clinical data follow-up and collection is lacking (17, 18, 30). HDRB boost techniques, which have been used for decades due to the introduction of afterloaders (9) instead provide the potential for analysis of toxicity and dosimetric data specific for the urethral side effects due to the routine inclusion of the urethral contour in the planning process.

This work has focused on fitting the LKB model parameters of urethral stricture data collected on a large cohort of patients treated with HDRB boost at one single institution for a time-period of 12 years. This NTCP model was created by considering urethrotomy recorded in the first 4 years after the treatment as an end-point. All toxicity data were prospectively recorded in a database and the follow-up was meticulously done by reviewing the patients at well set time intervals. Additionally, any correspondence with the treating doctors after brachytherapy was analyzed in order to look for additional urethrotomy recorded.

The predictive model fitted in this work showed a clear dose-effect relationship between the incidence of urethral stricture and the dose delivered to the urethra (Figure 2A), and it was obtained by using the DVH as opposed to a single representative dose parameter (for example, D10%). As shown in Table 1 by increasing the dose from 18 Gy in three fractions to 19 Gy in two fractions in a 2-day treatment schedule the incidence of strictures was increased by almost three times from an average rate of 6.9–20.5% for an identical cut-off time of 4 years of follow-up. This finding was previously documented by Hindson et al. (10) for a similar cohort of patients treated in the same institution, and it is here confirmed by means of a sigmoidal relationship. The fitted dose-response relationship showed that to ensure a toxicity rate to below 10% the urethral EUD should be limited to 85 Gy (with α/β = 5 Gy). Similar dose correlation was documented by other groups, with toxicity rates equivalent to the AHRO cohort for similar fractionation regimens (37, 38), and comparable follow-up time (5–6 years on average), however, comparisons were mainly performed by considering the prescription doses and not the planned dose to the urethra. In contrast, several publications reported no significant correlation in doses between the group that had toxicity and the group that did not have it (39, 40). For example, in the case of HDRB monotherapy, more recently in an analysis of 178 patients with a median follow-up time of 28.2 months, Tsang et al. (41) only reported 3% rate of urethral stricture and could not identify significant correlation with the toxicity and the urethral dose, identifying instead potential radiomics features that could predict the risk of developing toxicity on the pre-treatment MRI. This conclusion is similar to the work by Diez et al. (2) which instead considered a median follow-up time of 55 months for all groups. However, by fixing the follow-up time at 4 years the same authors reported an increase from 3 to 7% of the Kaplan–Meier estimates from the patients that were treated with 34 Gy in four fractions to the patients that were treated with 31.5 Gy in three fractions (14). Patients' follow-up time seems to represent an important variable in all of these studies, with large variations between groups and most works not considering a fixed time at which to compare different dose groups. For example, in a large cohort, Bece et al. (13) reported a decrease in toxicity rate from 12.8 to 3% by moving from 18 to 19 Gy in two fractions, however, the first group was followed for 4 years as opposed to 2 years for the second group limiting the information collected and the analysis. As shown in Table 1, in our group for a follow-up of 4 years the time to toxicity on-set decreased with increasing overall dose (from an average time of 3.6–2.1 years). So, a short follow-up time could potentially underestimate the recorded stricture rates.

The fitted model parameters (Table 2) well-represented the AHRO observed data, as shown by the calibration plot (Figure 2B), and internal calibration bootstrapped results (Figure 3). The AUC was of the order of 0.64, which is of the order of values obtained for most models based on dose features alone (42). An outlier was observed in the 80 Gy EUD dose group, believed to be associated with the little variability of urethral doses for patients in this dose group (Figures 2A,B). Notably, the volume parameter *n* was larger than expected (0.3), suggesting that the architecture of the urethra could be more parallel than generally believed, due to its shape and similarity to structures such as the spinal cord or the esophagus. However, this result could be related to the small volumes involved (ranging from 0.02 to 3.6 cm^3^ for the AHRO cohort), and the limitation in fitting the parameter with the data available. Additional studies have been undertaken in order to analyze surface or voxelized dose maps (18) of this organ, as opposed to the 2D representation provided by the DVH, to identify spatial and volumetric correlations with toxicity. In this work an α/β = 5 Gy was used in order to convert the physical doses into EQD_2_, and EUD. This value was chosen in accordance to work by Gloi and Buchanan (22) as representative of the urethral late effects, however, more dedicated studies are in progress in order to confirm the validity of this assumption, making it a limitation of this work.

The LKB model parameters were also tested by using data from a completely independent cohort treated with comparable HDRB boost doses to establish the generality of its predictive value. The external cohort was part of a large group of patients treated as part of the RADAR clinical trial (32) so all patients were planned by following a well-defined protocol for dose constraints and contouring guidelines. An interesting finding was the importance of urethra contouring in the assessment of NTCP dose-volume relationship. The RADAR patients' whole urethras were all initially contoured by the clinician as the lumen of the urinary catheter (here defined as original-Figure 1b, blue contour). The DVH extracted from this contour did not correlate with the initial model as shown in the calibration plot (Figure 4A). When re-outlined to match the AHRO contours (Figure 1a, yellow contour) the goodness of fit was confirmed. This result highlights that in order to understand the relationship between dose and toxicity, and compare the data of different groups, consensus for the outlining of the urethral volume is advisable, and contour practices should be clearly documented. It also suggests that in order to establish a dose-volume correlation the urethra should be contoured in order to include the urethral mucosal wall, and at least 10–20 mm of urethra distally to the prostate apex in order to include the bulbomembranous portion as previously highlighted (43). In this work for both AHRO and RADAR patients, the urethral dose provided by the external beam portion of the treatment was considered uniform and equivalent to the EBRT prescription dose. This method was followed due to the fact that for both cohorts the urethral structures were not contoured and considered at the time of treatment planning, and the plan was performed to achieve uniform PTV coverage (between 95 and 107% of the prescription dose). Due to the introduction of external beam hypofractionated treatments, and of routine urethral contouring this assumption might need to be modified in order to account for the available calculated urethral DVH information (35, 36).

In this work, the whole urethra was considered, as opposed to other studies (2, 41) in which the volume was divided in membranous and prostatic urethra.

Among the patients' clinical parameters, the effect of the use of neo-adjuvant androgen deprivation was investigated in the model fitting. Despite the modeling limitation that a small number of AHRO patients did not receive AD (Table 1), when LKB was fitted with and without AD, the TD50 (Gy) showed an absolute TD50 reduction of 13.3 Gy without AD, suggesting that AD could act as dose-modifier and a protective effect on urethral toxicity. A similar result was previously documented by Palorini et al. (30) for a large multicenter group of patients treated with EBRT, and it could be due to the known effect of tumor shrinkage and reduction of the irradiation volume, and potentially a cytoreductive effect (30, 44).

All DVHs used in this study were extracted from the treatment planning system and so they are representative of the planned dose. This is a known limitation as experience and previous works (10, 13) have shown the potential for prostate swelling and needle movement with respect to the anatomy, which could potentially under or overestimate the dose-toxicity correlation found. As of 2017, our group has started performing on-line verification between CT and treatment and re-scanning and planning the patients when the movement exceeds our clinical tolerances (45), and data will be analyzed when mature.

## Conclusion

Urethral toxicity is a limiting factor in providing additional dose escalation in radiotherapy of the prostate. For HDRB of prostate cancer clear urethral dose guidelines are still not available due to the variety of dose prescription used and the variety of contouring protocols. In this work, an LKB model was fitted to the risk of urethral stricture for a large single center cohort. The model was then externally validated with independent patients' clinical and dosimetric data, showing a clear and reproducible relationship between dose delivered to the whole organ and urethral toxicity. When clinical factors were included findings showed that for patients that did not receive neo-adjuvant androgen deprivation, model fitting provided a lower TD50 (Gy) suggesting a protective effect on urethral toxicity, as previously highlighted for EBRT studies.

## Data Availability Statement

The datasets generated for this study will not be made publicly available due to the ethics considerations.

## Ethics Statement

The studies involving human participants were reviewed and approved by Alfred Hospital Ethics Committee (Project 323/14). Use of RADAR data was approved by the TROG Publications Committee. Written informed consent for participation was not required for this study in accordance with the national legislation and the institutional requirements.

## Author Contributions

VP has designed the study, collected the data, performed the MATLAB calculations described in the methods, and has written the draft of the manuscript. TR has provided extensive technical advice on the implementation of the work, model building, and validation and has revised several versions of both calculations and manuscript. EO has provided the MATLAB code used in the study and has provided advice on the technical component. ME, JD, DJ, and AS have provided the RADAR data used for the external validation and JM has provided advice and patients data used for model building. All co-authors contributed to proof-reading of the manuscript.

## Conflict of Interest

DJ was employed by GenesisCare and 5D Clinics. ME has an honorary position at 5D Clinics. DJ and ME each have a financial shareholding in 5D Clinics. The remaining authors declare that the research was conducted in the absence of any commercial or financial relationships that could be construed as a potential conflict of interest.
